# Analysis of the current status and influencing factors of nurses’ patient safety competence: a potential profile analysis

**DOI:** 10.3389/fmed.2024.1481261

**Published:** 2024-10-23

**Authors:** Qian Xiao, Xi Huang, Xin Ren, Xiulin Wen, Yujian Zhao

**Affiliations:** ^1^Department of Nursing, Xijing Hospital, Air Force Medical University, Xi’an, China; ^2^Department of Nursing, First Affiliated Hospital of Xi'an Jiaotong University, Xi’an, China

**Keywords:** nurses, patient safety competency, potential profile analysis, cross-sectional survey, latent profile analysis

## Abstract

**Objective:**

The study aimed to understand the current situation of hospital nurses’ patient safety competence, identify potential categories, and analyze the distribution characteristics of demographic variables in each subgroup.

**Methods:**

A total of 1,996 nursing staff were selected to be included in this research using a convenience sampling method. A questionnaire survey was conducted using the Chinese version of the Patient Safety Ability Scale, and three dimensions of the Patient Safety Ability Rating Scale were used as explicit variables for potential profile analysis. The influencing factors of different potential profiles of nurses’ patient safety ability were analyzed using logistic regression analysis.

**Results:**

The safety ability of the 1,996 nurses could be categorized into three potential groups as follows: “ability risk group” (14.3%), “medium ability group” (32.9%), and “ability advantage group” (52.7%), with monthly income, participation in department training, self-learning, and love of nursing as the influencing factors.

**Conclusion:**

The overall patient safety ability of nurses is at an upper-middle level. The classification of nurses can be predicted by factors, such as monthly income, participation in department training, self-directed learning, and love of nursing. Nursing managers can formulate targeted interventions according to the influencing factors of potential profiles to improve nurses’ patient safety ability.

## 1 Introduction

The Action Plan for Comprehensive Healthcare Quality Improvement (2023–2025) places special patient safety initiatives high on the agenda (1). Patient safety is the cornerstone of healthcare quality, and achieving “zero harm” is the ultimate goal of patient safety (2). Nurses, as an important part of the healthcare team, are key players in providing safe care to patients, and their ability to achieve patient safety is important for improving the quality of care and patient health outcomes (3).

Patient safety competence refers to the attitudes, knowledge, and skills that nurses need to reduce the risk of adverse events during a patient’s hospitalization (4). Currently, research on patient safety by foreign scholars is maturing (5). However, in China, research on this topic began later and remains in the exploration stage (6). In the current field of cross-sectional studies, many studies tend to focus on relatively single-level influencing factors, including individual factors, such as sex and age, and economic factors, such as monthly income (7–9). The results of these studies primarily rely on scores obtained from various scales. However, it is worth noting that these studies have largely failed to conduct in-depth analysis of the differences in population characteristics across different score levels (such as low, medium, and high scores). From a methodological perspective, this type of single-level factor research design limits our ability to fully understand and analyze the study subjects. In practice, different score levels often reflect variations in how populations perform on specific dimensions, and these differences may be linked to various underlying factors. Therefore, without thoroughly investigating the population characteristics associated with different score levels, we may overlook important information, which could compromise the accuracy and validity of the study results.

Latent Profile Analysis (LPA), as an individual-centered statistical analysis method, can identify subgroups with common level patterns based on an individual’s selection pattern of dominant variables. This approach further captures the characteristics of the population that cannot be observed through variable-centered statistics (10). In light of this, this study used the latent profile analysis to identify potential categories of nurses’ patient safety competence and analyze the differences in population characteristics across categories, aiming to provide a reference for improving nurses’ patient safety competence.

## 2 Materials and methods

### 2.1 Study participants

To participate in the study, individuals must meet the following inclusion criteria: they must hold a nursing practice certificate, have a minimum of 1 year of clinical nursing experience, and provide informed consent along with agreeing to participate voluntarily. Those who will be excluded from the study include individuals who have been away from clinical work for 3 months or more, whether due to leave, advanced study, or external training. Furthermore, participants will be eliminated from the study if they complete the questionnaire in less than 300 s, if their responses follow a clear pattern, or if there are significant logical inconsistencies in their answers throughout the questionnaire.

According to Kendall’s sample size estimation method, the sample size should be 5 to 10 times the number of independent variables, while accounting for 20% of invalid questionnaires. In this study, a total of 54 variables were included and the required sample size ranged from 281 to 551, which meets the estimation requirements.

### 2.2 Survey instruments

#### 2.2.1 General information questionnaire

A general information questionnaire was designed independently. It mainly included 15 aspects, such as age, sex, education, hospital grade, title, department, average number of night shifts per month, average time of direct contact with patients per day, number of overtime hours per week, mode of employment, and monthly income.

#### 2.2.2 Patient safety competency self-evaluation

The patient safety competency self-evaluation (PSCSE) tool was revised by Lee (11) and translated and localized by Li Ling (12). The scale consists of 39 entries across three dimensions, including knowledge of patient safety culture (4 entries), error detection and response (12 entries), and error prevention strategies and professional responsibilities (23 entries). Each item is rated on a 5-point Likert scale, where a score of 1–5 indicates “not at all compliant,” “not compliant,” “sometimes compliant,” “mostly compliant,” and “completely compliant.” The total score for the scale is 195; the higher the score, the better the nurse’s competence in patient safety. Cronbach’s alpha coefficient for the scale was 0.985, while Cronbach’s alpha coefficients for the dimensions ranged from 0.951 to 0.983.

### 2.3 Data collection methods

In June 2022, the researchers sent recruitment invitations and informed consent forms via email to the Shaanxi Province Nursing Quality Control Center and contacted the nursing departments of municipal hospitals in Xi’an, Shaanxi Province. The prepared instructions and questionnaire link^1^ were distributed to clinical nurses through the Wenjuanxing app. The questionnaire was administered anonymously. The participants were required to complete all questions before submission, with each person allowed to submit only once, ensuring the completeness and validity of the questionnaire. After the questionnaires were collected, two researchers reviewed the data and manually eliminated invalid questionnaires. A total of 2,130 questionnaires were distributed, of which 134 invalid questionnaires were excluded and 1996 valid questionnaires were recovered, with an effective recovery rate of 93.7%.

### 2.4 Ethical considerations

Before data collection, the investigators explained the purpose, process, and potential benefits and risks to the head of the Shaanxi Province Quality Control Center and the respondents. The study complied with the provisions and ethical principles of the 1995 Declaration of Helsinki (revised in Edinburgh in 2000). This study was approved by the Ethics Committee of the First Affiliated Hospital of Xi’an Jiaotong University (No. XJTUIAF2023LSK-305).

### 2.5 Statistical analysis

#### 2.5.1 Descriptive statistics

First, we used SPSS 22.0 to tabulate and process the data. The quantitative data that followed a normal distribution were presented as mean ± standard deviation (x ± s), while the qualitative data were expressed as frequency and percentage (%). We analyzed the demographic data of the 1,996 participants and found no missing items. Descriptive statistics were then conducted for the dependent and independent variables, and the corresponding statistical values and *p*-values were calculated. See Table 1 for details.

**Table 1:** General information of the nurses and the univariate analysis of the potential categories of patient safety competence among the nurses with different characteristics.

Sports event	Categorization	Capacity Risk Group (*n* = 287)	Intermediate competence group (*n* = 656)	Competence Advantage Group (*n* = 1,053)	t/*F* value	*p*-value
Distinguishing between the sexes	Male	7 (2.4)	16 (2.4)	31 (2.9)	0.482	0.786
	Female	280 (97.6)	640 (97.6)	1,022 (97.1)		
Age	<30 years	112 (39.0)	236 (36.0)	392 (37.2)	5.720	0.455
	30–40 years old	148 (51.6)	323 (49.2)	532 (50.5)		
	41–50 years	24 (8.4)	85 (13.0)	112 (10.6)		
	>50 years old	3 (1.0)	12 (1.8)	17 (1.6)		
Education	Vocational secondary school	1 (0.3)	14 (2.1)	9 (0.9)	9.314	0.157
	Three-year college	96 (33.4)	203 (30.9)	358 (34.0)		
	Undergraduate (adjective)	186 (64.8)	429 (65.4)	674 (64.0)		
	Master’s degree or above	4 (1.4)	10 (1.5)	12 (1.1)		
Hospital level	Grade 3A	115 (40.1)	314 (47.9)	504 (47.9)	15.652	0.208
	Grade 3B	9 (3.1)	13 (2.0)	16 (1.5)		
	Grade C	0 (0.0)	1 (0.2)	1 (0.1)		
	Grade 2A	150 (52.3)	312 (47.6)	497 (47.2)		
	Grade 2B	10 (3.5)	16 (2.4)	26 (2.5)		
	Class A(B)	0 (0.0)	0 (0.0)	1 (0.1)		
	Currently unknown	3 (1.0)	0 (0.0)	8 (0.8)		
Mode of employment	Be on staff	54 (18.8)	142 (21.6)	216 (20.5)	0.999	0.607
	Employment system	233 (81.2)	514 (78.4)	837 (79.5)		
Monthly salary	<¥5,000	224 (78.0)	426 (64.9)	688 (65.3)	22.930	<0.001
	¥5,000 ~ 10,000	60 (20.9)	219 (33.4)	332 (31.5)		
	>¥10,000	3 (1.0)	11 (1.7)	33 (3.1)		
Administrative division	General medicine	82 (28.6)	207 (31.6)	341 (32.4)	14.973	0.380
	Neurosurgery	68 (23.7)	147 (22.4)	221 (21.0)		
	Department of gynecology and obstetrics	18 (6.3)	51 (7.8)	82 (7.8)		
	Gynecology	24 (8.4)	59 (9.0)	115 (10.9)		
	ICU	10 (3.5)	29 (4.4)	43 (4.1)		
	Operating rooms	10 (3.5)	32 (4.9)	37 (3.5)		
	Emergency call	21 (7.3)	40 (6.1)	47 (4.5)		
	(sth. or sb) Else	54 (18.8)	91 (13.9)	167 (15.9)		
Title	Physiotherapists	67 (23.3)	144 (22.0)	240 (22.8)	13.950	0.030
	Physiotherapists	138 (48.1)	273 (41.6)	484 (46.0)		
	Nurse practitioner-in-charge	77 (26.8)	202 (30.8)	270 (25.6)		
	Associate Nurse Practitioner and above	5 (1.7)	37 (5.6)	59 (5.6)		
Years of experience	<5 years	65 (22.6)	161 (24.5)	235 (22.3)	6.441	0.376
	5 to 10 years	102 (35.5)	216 (32.9)	372 (35.3)		
	>10 years ~ <15 years	77 (26.8)	146 (22.3)	251 (23.8)		
	≥15 years	43 (15.5)	133 (20.3)	195 (18.5)		
Average patient contact time per day (h)	<5	19 (6.6)	43 (6.6)	66 (6.3)	4.153	0.386
	5 ~ 8 h	116 (40.4)	293 (44.7)	422 (40.1)		
	>8	152 (53.0)	320 (48.8)	565 (53.7)		
Average number of night shifts per month	<5	118 (41.1)	290 (44.2)	488 (46.3)	2.676	0.262
	≥5	169 (58.9)	366 (55.8)	565 (53.7)		
Overtime per week	<3	169 (58.9)	411 (62.7)	680 (64.6)	3.574	0.467
	3 ~ 5 times	75 (26.1)	156 (23.8)	245 (23.3)		
	>5	43 (15.0)	89 (13.6)	128 (12.2)		
Date of most recent participation in departmentally organized training in patient safety competency	Within a month	191 (66.6)	448 (68.3)	865 (82.1)	83.376	<0.001
	1 ~ 6 months	40 (13.9)	130 (19.8)	127 (12.1)		
	>6 months	24 (8.4)	37 (5.6)	38 (3.6)		
	never before	32 (11.1)	41 (6.3)	23 (2.2)		
Time of last active review of literature or books on patient safety competence	Within a month	142 (49.5)	335 (51.1)	722 (68.6)	97.727	<0.001
	1 ~ 6 months	58 (20.2)	150 (22.9)	206 (19.6)		
	>6 months	33 (11.5)	77 (11.7)	65 (6.2)		
	Never before	54 (18.8)	94 (14.3)	60 (5.7)		
Do you love nursing?	Yes	212 (73.9)	560 (85.4)	1,003 (95.3)	117.332	<0.001
	Clogged	75 (26.1)	96 (14.6)	50 (4.7)		

#### 2.5.2 Latent profile analysis

In the medical field, latent profile analysis (LPA) is used to identify heterogeneous subgroups within a population. This method categorizes individuals into different groups, such as low-functioning and high-functioning groups, based on scores from outcome measures. It optimizes hospital management systems, leading to more rational and efficient allocation of medical resources. This approach provides strong support and assistance for patient classification management and medical quality improvement in hospitals.

Next, we used Mplus 8.3 software to conduct the latent profile analysis. The LPA classified the individuals based on their self-reported levels of nurses’ patient safety competency, as measured by the PSCSE. Specifically, our analysis was based on the PSCSE dimensions (e.g., knowledge, skills, and attitudes) and the overall score, as detailed in Table 2.

**Table 2:** Nurses’ patient safety competency scores.

	Score	Entry parity (accountancy)
Knowledge of safety culture	15.54 ± 3.81	3.88 ± 0.02
Error detection and response	49.38 ± 9.52	4.12 ± 0.01
Error prevention strategies and professional responsibilities	102.70 ± 16.68	4.47 ± 0.01
Patient safety competency scale	167.62 ± 27.33	4.30 ± 0.02

We provided a comprehensive overview of various parameterized models in LPA. The model fit indexes used to evaluate these models included the Akaike information criterion (AIC), Bayesian information criterion (BIC), and sample-adjusted BIC (aBIC). Lower values of these indexes indicate a better model fit. In addition, information entropy was used to assess classification accuracy, with an entropy value of 0.6 indicating approximately 20% error in classification and an entropy value of approximately0.8 indicating over 90% classification accuracy.

**Figure 1:**
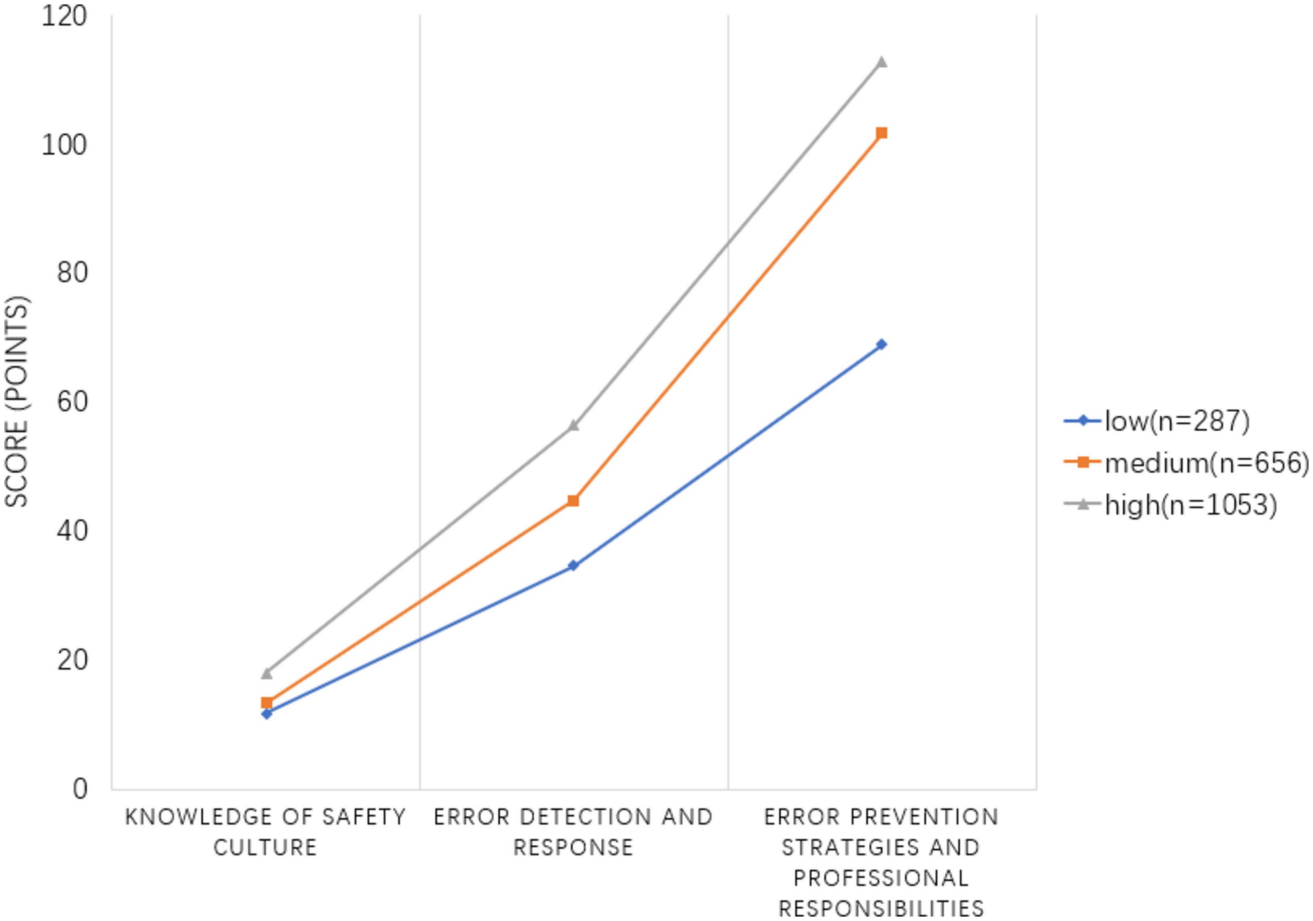
Potential profile characteristics of the three dimensions of nurses’ patient safety competency.

Moreover, Mplus offers two likelihood ratio test indicators: the Lo–Mendell–Rubin adjusted likelihood ratio test (LMR-LRT) and the bootstrap likelihood ratio test (BLRT). These indicators are used to compare the fit of latent class models. We assessed fit indexes and model solutions from various parameterized methods, considering variance and covariance changes across multiple models. If the *p*-values for these two tests were less than 0.05, it indicated that the k-class model was significantly better than the K-1 class model. During model selection, we also considered the variability in classification measures and the number of individual profiles, typically ≥10%. This information is detailed in Figure 1 and Tables 3–4, which summarize the fit indexes for each examined model.

**Table 3:** Comparison of the potential profile model fit metrics (*n* = 1996).

Mould	AIC	BIC	aBIC	Entropy (physics)	*p*- value		Categorical probability
					LMR-LRT	BLRT	
1	42575.558	42609.151	42590.089	–	–	–	
2	40176.833	40232.822	40201.051	0.919	<0.001	<0.001	0.19890/0.80110
3	39069.437	39147.822	39103.343	0.864	<0.001	<0.001	0.14379/0.32866/0.52756
4	38176.581	38277.361	38220.174	0.916	<0.001	<0.001	0.01353/0.13928/0.53657/0.31062
5	37447.392	37570.568	37500.673	0.906	0.005	<0.001	0.12625/0.01303/0.17084/0.19038/0.49950
6	37298.798	37444.370	37361.766	0.907	0.105	<0.001	0.10822/0.01253/0.15631/0.49699/0.04058/0.18537

**Table 4:** Comparison of the scores across the dimensions of patient safety competence among the nurses in three potential categories (x ± s).

Sports event	Capacity risk group (*n* = 287)	Intermediate competence group (*n* = 656)	Competence Advantage Group (*n* = 1,053)	*F*-value	p- value
Dimension 1	11.68 ± 2.94	13.22 ± 3.29	18.03 ± 2.21	970.478	<0.001
Dimension 2	34.66 ± 6.90	44.47 ± 5.49	56.46 ± 4.00	2595.200	<0.001
Dimension 3	68.59 ± 12.41	101.17 ± 8.97	112.95 ± 3.35	4093.545	<0.001
Totals	114.92 ± 19.40	158.87 ± 10.36	187.43 ± 7.02	5464.866	<0.001

Finally, to predict outcomes and explain the interactions between independent variables and their effects on dependent variables, we conducted a logistic regression analysis with the three latent classes of nurses’ patient safety competency as the dependent variable and the statistically significant factors from one-way ANOVA as the independent variables. Details of this analysis are presented in Table 5.

**Table 5:** Logistic regression analysis of the factors influencing different potential categories of the nurses’ patient safety competence (*n* = 1996).

Variant	Β-value	Standard error	Wald *χ*^2^ value	*p*- value	OR (95% CI)
Monthly salary					
<¥5,000	−1.209	0.338	12.804	<0.001	0.298 (0.154 ~ 0.579)
¥5,000 ~ 10,000	−0.819	0.336	5.933	0.015	0.441 (0.228 ~ 0.852)
>¥10,000^a^	–	–	–	–	–
Title					
Physiotherapists	0.028	0.241	0.014	0.907	1.029 (0.641 ~ 1.651)
Physiotherapists	0.051	0.228	0.050	0.823	1.052 (0.673–1.646)
Nurse practitioner-in-charge	−0.174	0.227	0.592	0.441	0.840 (0.539 ~ 1.310)
Associate Nurse Practitioner and above^a^	–	–	–	–	–
Trained in safety					
Within a month	0.742	0.236	9.881	0.002	2.101 (1.322 ~ 3.338)
1 ~ 6 months	0.445	0.252	3.105	0.078	1.560 (0.951 ~ 2.559)
>6 months	0.274	0.287	0.910	0.340	1.316 (0.749 ~ 2.311)
Never before^a^	–	–	–	–	–
Proactive review of literature and books					
Within a month	0.754	0.175	18.563	<0.001	2.126 (1.509 ~ 2.996)
1 ~ 6 months	0.415	0.186	4.967	0.026	1.514 (1.051 ~ 2.180)
>6 months	0.008	0.210	0.001	0.971	1.008 (0.668 ~ 1.521)
Never before^a^	–	–	–	–	–
Do you love nursing?					
Yes	1.254	0.139	81.492	<0.001	3.506 (2.670 ~ 4.603)
Clogged^a^	–	–	-	–	–

^a represents the reference group.^

## 3 Results

### 3.1 General information of the nurses

#### 3.1.1 Nurses’ patient safety competency scores

The total patient safety competency score of the 1,996 nurses was 167.62 ± 27.33, which suggested that the nurses’ patient safety competency was at a medium to high level, as shown in Table 2.

#### 3.1.2 Results and naming of the potential profiles of nurses’ patient safety competency

The potential profile analysis of nurses’ patient safety competency across the three dimensions of the expectation score aimed to establish a 1–6 potential category model. Each model fit index is detailed in Table 3. With the increase in the number of categories, the entropy value exceeds 0.8, while the values of the AIC, BIC, and aBIC decrease. However, the LMR-LRT value for the six categories (*p* = 0.105) is not significant, suggesting that the model lacks a good fit. Although the values of the AIC, BIC, and aBIC show a decreasing trend with the increase in the number of classifications and the entropy value of the four-classification model is higher than that of the three-classification model, the uneven distribution of category probabilities in the four-classifications model—where one group accounts for only 1.3% of the total—led to the conclusion that, based on the comprehensive results of the statistical analysis and theoretical considerations (13), model 3 was the best potential profile model. Based on the distribution of conditional means on the three dimensions in Model 3, see Figure 1, the categories were named according to the epiphenomenal characteristics of the dimensions of the scale. They were designated as follows: the “ability risk group” with 287 people, accounting for 14.3%; the “medium ability group” with 656 people, accounting for 32.3%; and the “competence advantage group” with 1,053 people, accounting for 52.7%. The differences in the scores of the three potential profiles in the three dimensions were statistically significant (*p* < 0.001), as indicated by one-way ANOVA, as shown in Table 4.

#### 3.1.3 Univariate analysis of the potential categories of patient safety competence for the nurses with different characteristics

The results showed that the differences between the three potential categories were statistically significant (*p* < 0.05) in terms of monthly income, title, time spent attending training in the department, time spent actively reviewing literature or books, and whether or not they loved their nursing position. See Table 1 for details.

#### 3.1.4 Multifactor logistic regression analysis of the potential profiles of nurses’ patient safety competence

The logistic regression analysis of the three potential categories of nurses’ patient safety competence as the dependent variables and the statistically significant factors from one-way ANOVA as the independent variables showed that a monthly income of <¥5,000 (OR = 0.298) was a risk factor for nurses’ patient safety competence. In contrast, receiving safety training within a month (OR = 2.101), actively reviewing literature and books within a month (OR = 2.126), and possessing a strong affection for the nursing position (OR = 3.506) were identified as protective factors for nurses’ patient safety competence. See Table 5 for details.

## 4 Discussion

### 4.1 Characterization of the current and potential categories of nurses’ patient safety competence

The nurses’ patient safety competence score was 167.62 ± 27.33, which could be divided into the competence risk group (14.3%), medium competence group (32.9%), and competence advantage group (52.7%) based on the potential characteristics of the individual respondents. This suggested that there was significant individual variability in the nurses’ patient safety competence. The “competence advantage group” had the most representative samples, which were mainly characterized by strong nursing practice abilities, the ability to accurately identify unsafe factors, the active implementation of effective preventive measures to avoid the occurrence of errors, and the reduction of injury risks during the hospitalization of patients (14). The total percentage of the nurses in the “competence risk group” and “medium competence group” was 47.3%. The nurses in these groups exhibited low patient safety competence. These Nurses fail to systematically master nursing safety knowledge and skills. In clinical practice, they have a low awareness of recognizing potential dangers, insufficient experience in coping with risks, and ineffective risk prevention measures. The scores of these nurses were low across all dimensions, especially in the dimensions of “knowledge of safety culture” (15.54 ± 3.81) and “error detection and response” (49.38 ± 9.52). This reflects a need for the nurses in this group to strengthen their knowledge and abilities related to patient safety, identifying them as a key population for intervention. This finding is consistent with the results of Qian et al. (7). Compared to traditional studies of nurses’ patient safety competence, the categorization in this study not only helps determine the levels of nurses’ scores but also helps identify the characteristics of different categories of nurses to better target the implementation of management measures. At present, to better promote patient safety and meet the evolving needs of the times, hospitals have established higher requirements for nurses’ patient safety competence. Given the insufficiency of patient safety education, it is particularly urgent to establish a complete content system for patient safety knowledge training (15). The focus of nursing managers will be to explore how nurses can improve their knowledge related to safety culture. At the same time, they need to examine how to effectively implement patient safety education and what training methods should be adopted. Therefore, nursing managers should assess the safety competence of nurses in a timely manner, identify nurses with low levels of patient safety competence as early as possible, and then explore the reasons for the low scores in depth, so as to scientifically formulate precise, feasible, operationally effective, and targeted intervention methods. Additionally, they can also leverage the positive guiding and peer support role of nurses in the “competence advantage group” to enhance the overall level of nursing practice.

### 4.2 Monthly income as a risk factor for nurses’ patient safety competence

The percentage of nurses with a monthly income of <¥5,000 in the “competence risk group” was 78%, while 3.1% of the nurses in the “competence advantage group” had a monthly income of >¥10,000, which was significantly higher than that of the other groups. This is similar to the findings of Ying et al. (16) The results of the survey conducted among nurses in 13 tertiary hospitals in Shanghai are consistent. The reason for this is that, firstly, remuneration reflects, to a certain extent, the level of labor capacity and the quality of nursing services among different nursing staff. Therefore, new nurses with higher monthly incomes are more likely to belong to the “ability advantage type.” Secondly, performance appraisal is a modern incentive system management method that can enhance nurses’ motivation toward work through the distribution of performance rewards, which strengthens nurses’ sense of belonging, increases their commitment to work, and then improves their safety awareness and behavior (17). On the contrary, the mismatch of pay levels is difficult to address. This mismatch can hardly allow nurses to improve their subjective motivation regarding safety ability. Therefore, nursing managers should provide more reasonable and optimized suggestions to the hospital operations department for nurses’ performance allocation, such as performance appraisal indexes. In addition to the common indexes of title, education, and years of working experience, it is recommended to include nursing safety competence, job competence, and quality of nursing care as reference indexes of performance appraisal. Furthermore, the appraisal results should be linked to nurses’ renewed employment, salary, and bonuses, as well as special post allowances, bonus factors, opportunities for outbound training, and initiatives to improve the safety awareness and behavior of nurses. The results of the assessment should be linked to the renewal of employment, salary, and bonuses of nurses. In addition, aspects such as special post allowances, bonus coefficients, training opportunities, and promotion titles should be adjusted to fully mobilize the motivation and internal drive of nursing staff, ensuring a collective effort toward patient safety (18). We will fully mobilize the motivation and internal drive of nursing staff and work together to ensure patient safety.

### 4.3 Having received safety training, proactive review of literature and books, and having a love for the nursing position are protective factors for nurses’ patient safety competence

The logistic regression results showed that the proportion of the nurses who received safety training within a month was as high as 68.6% in the “Competence Advantage Group.” In addition, the probability of error risk for the nurses who received the training decreased by 2.101 times compared to those who never received the training. This suggests that nurses’ competence in patient safety is directly proportional to the duration of the training and that there is a significant difference in nurses’ competence in patient safety based on the duration of the training. This is consistent with the findings of Taoyuan et al. (8). Safety education and training have a crucial impact on nurses’ career development, and it is important to explore new training methods (19, 20). After 6 months of nursing safety training for 56 new nurses using the “action learning method,” the nurses’ safety attitudes, operational skills, and comprehensive clinical abilities were higher than those of the conventional group (21). A microteaching model for nursing safety education has yielded positive results for nurses. Shujuan et al. (22) demonstrated that the use of mind mapping combined with case analysis can help nurses comprehensively analyze their conditions, make effective judgments, and make correct nursing decisions in complex scenarios. Some scholars have also been exploring new forms of teaching, including Hongjuan et al. (23). Based on an analysis and summary of adverse events that have occurred in recent years, they have prepared scenario teaching scripts and adopted a scenario simulation teaching method, which effectively strengthen nurses’ safety behaviors and enhance their safety competence. In light of this, nursing managers should develop a graded training program to enhance nurses’ safety competence and regularly conduct special continuing education training and assessment. Furthermore, they should also consider incorporating microteaching, mind mapping combined with case analysis, and situational simulation teaching on the basis of the traditional training forms.

The results of this study showed that the nurses who actively consulted books within a month (68.6%) were more likely to develop into the “competence advantage group” than the nurses who had never initiated independent study (5.7%). This finding is consistent with the findings of Ouyang and Lei (6) and Xi et al. (24), which indicated that the greater the ability to learn independently, the higher the nurses’ patient safety competency scores. As nurses spend most of their time providing direct care to patients, they are required to be highly competent in their roles. However, the development of nurses’ competence not only requires input from clinical safety education but also relies on continuous and effective self-directed learning in the workplace. Self-directed learning is an important part of a nurse’s overall quality of life and is a key means of developing the skills needed for a professional career. Some studies have shown that (25) the higher the independent learning ability of nurses, the easier it is for them to move away from the traditional passive learning mode in the clinical field. This allows them to assume a leadership role and take initiative, fully utilizing the knowledge and skills they have acquired to cope with the nursing work, which will improve their nursing safety ability in the long run. It can be seen that the acquisition of knowledge, skills, and attitudes for patient safety lies not only in the improvement of education but also focuses on individual efforts, where the motivation to learn is especially important for stimulating and maintaining the practice of learning behaviors (26).

Compared to the nurses who did not love their nursing position, the nurses who love it had the highest percentage (95.3%) in the “competence advantage group,” which was consistent with the results of Yang et al.’s study (27). The higher patient safety competence scores of the nurses who love their nursing position are mainly due to the fact that nurses who identify with their profession tend to promote positive self-perception, which in turn improves their evaluation of their knowledge and skills (28). Furthermore, nurses who are more loyal to their nursing position tend to be more committed to their work and have stronger self-efficacy. Driven by a sense of responsibility and mission, they are more likely to understand the meaning of their work and their job roles, which directly affects the quality of care they provide to patients. Therefore, it is recommended that nursing managers provide nurses with positive practice planning guidance, increase their interest in learning, and use appropriate authorization based on individual abilities. In addition, they should address differences in job placement and promotion and provide more career development paths to enhance their sense of professional identity, thereby improving their work ability.

### 4.4 Limitations of this study

Firstly, the questionnaire for this study was distributed via Wenjuanxing in June 2022. This timing might have coincided with peak or off-peak periods in certain departments, potentially affecting the respondents’ answers and not fully reflecting the situation throughout the study period. In addition, internal organizational changes, such as policy adjustments and staff turnover, might have also influenced the respondents’ responses.

Secondly, the results of this study are based on a sample from a specific region in China, which might have exhibited regional and cultural differences due to variations in healthcare systems and environments. These regional and cultural differences might limit the generalizability of the findings to other areas or countries with different healthcare systems. We acknowledge this limitation as the study does not achieve ideal diversity in terms of nationality, ethnicity, or race.

Lastly, we recognize that latent profile analysis is just one of several methods for identifying profiles or clusters within a population. In addition to the LPA used in this study, other methods such as hierarchical cluster analysis, two-factor models, and exploratory structural equation modeling can also be used to derive profiles. This study was conducted solely through a cross-sectional survey, lacking depth and breadth. We recommend that future research expand the field by combining cross-sectional and longitudinal approaches, as well as integrating qualitative and quantitative research methods for a more comprehensive investigation of patient safety competence.

## 5 Summary

In summary, the overall patient safety competence of the nurses was at a moderate to high level, and the patient safety competence of the nurses could be categorized into three groups: competence risk group, moderate competence group, and competence advantage group. The competence risk group needs to improve in all aspects, especially in the dimensions of “knowledge of safety culture” and “error detection and response.” Nurses’ monthly income, safety training, self-directed literature review, and love of nursing were the factors influencing the different potential categories of nurses’ patient safety competence. Nursing managers should implement targeted measures to improve patient safety competence based on these influencing factors.

## Data Availability

The raw data supporting the conclusions of this article will be made available by the authors, without undue reservation.

## Footnotes

^1^ https://www.wjx.cn/vm/e5voaUX.aspx
